# Gentamicin and Citronella-Derived Compounds as Dual Modulators of Inflammation-Associated Targets in Acne Vulgaris

**DOI:** 10.3390/ijms262110628

**Published:** 2025-10-31

**Authors:** Adeola Tawakalitu Kola-Mustapha, Muhabat Adeola Raji, Samah H. O. Zarroug, George Oche Ambrose

**Affiliations:** 1 Department of Pharmaceutical Sciences, College of Pharmacy, Alfaisal University, Riyadh 11533, Saudi Arabia; 2 Department of Microbiology and Immunology, College of Medicine, Alfaisal University, Riyadh 11533, Saudi Arabia; mraji@alfaisal.edu; 3 Department of Pharmacology and Therapeutics, College of Medicine, Alfaisal University, Riyadh 11533, Saudi Arabia; szarroug@alfaisal.edu; 4 Department of Molecular Biology, Centre for Malaria and Other Tropical Diseases Care, University of Ilorin Teaching Hospital, Ilorin 240101, Nigeria; ocheab1@gmail.com

**Keywords:** acne vulgaris, SPTBN1, SIPA1L1, isotretinoin, MD simulation

## Abstract

Acne Vulgaris is a chronic inflammatory skin disorder driven by a combination of microbial colonization, immune dysregulation, and disruption of the epidermal barrier. Although isotretinoin remains the most effective treatment, the molecular mechanisms underlying its anti-inflammatory effects are incompletely understood. This study integrates transcriptomic meta-analysis and computational drug screening to identify novel therapeutic targets and candidate compounds for acne management. Three publicly available GEO datasets (GSE6475, GSE10433, GSE11792) were analyzed to identify differentially expressed genes (DEGs) associated with isotretinoin response. Among these, Spectrin beta, non-erythrocytic 1 (SPTBN1) and Signal-induced proliferation-associated 1-like protein 1 (SIPA1L1) emerged as consistently regulated genes with known roles in cytoskeletal organization and immune signaling, respectively. To assess the druggability of these targets, molecular docking was conducted using gentamicin and natural compounds derived from *Cymbopogon winterianus*, including citronellol, citral, citronellal, and geraniol. Gentamicin demonstrated the strongest binding affinity to SIPA1L1 (−8.6 kcal/mol) and SPTBN1 (−5.9 kcal/mol), forming multiple hydrogen bonds and hydrophobic contacts. Subsequent 100 ns molecular dynamics (MD) simulations confirmed the stability of the gentamicin–protein complexes, as evidenced by favorable RMSD, RMSF, and energy profiles. Interaction energy decomposition revealed strong contributions from electrostatic and van der Waals forces. These findings highlight the potential of gentamicin, and possibly structurally related natural compounds, as modulators of host inflammatory pathways implicated in acne. The study further underscores the utility of integrating transcriptomics, molecular docking, and MD simulation for early-phase therapeutic discovery targeting inflammation and barrier dysfunction in dermatological diseases.

## 1. Introduction

Acne Vulgaris, a prevalent chronic skin disorder, is primarily attributed to the colonization of hair follicles by *Cutibacterium acnes* (formerly *Propionibacterium acnes*) [1]. This bacterium contributes significantly to the inflammatory cascade underlying acne lesions through the activation of innate immune responses and secretion of virulence factors [2]. The emergence of antibiotic-resistant *C. acnes* strains, coupled with the limited efficacy and side effects of current therapies, highlights the urgent need for novel therapeutic interventions [3]. In this context, the present study explores the potential of natural compounds derived from *Cymbopogon winterianus* (citronella) to serve as alternative or complementary inhibitors of acne-associated targets.

*C. winterianus* is well known for its antimicrobial properties, largely attributed to its essential oil constituents including citronellol, citral, citronellal, and geraniol. These compounds have demonstrated inhibitory activity against a broad spectrum of bacteria and fungi, including *C. acnes*, with minimum inhibitory concentrations (MICs) ranging from 312.5 to 625 µg/mL [4].

Given the role of *C. acnes* surface sialidase in facilitating tissue invasion and immune evasion [5], the present study initially sought to target this enzyme using phytochemicals from *C. winterianus* through molecular docking analysis. However, we extended the investigation to include transcriptome-based identification of host anti-inflammatory targets modulated during isotretinoin therapy—specifically SPTBN1 and SIPA1L1—which play key roles in skin barrier regulation and immune signaling.

This study allowed for a more host-centered therapeutic strategy, investigating whether *C. winterianus*-derived compounds and gentamicin could also bind to and stabilize these proteins, using molecular docking and 100-nanosecond molecular dynamics (MD) simulations. This integrated computational approach aims to elucidate the molecular interactions, binding affinities, and dynamic behavior of candidate compounds, offering a dual-pathway strategy—targeting both microbial virulence and host inflammation.

The study’s dual focus—on both bacterial virulence factors and host gene targets—supports the development of multifaceted treatment strategies that align with the global imperative to combat antibiotic resistance while leveraging natural products in dermatological drug discovery.

## 2. Results and Discussion

### 2.1. Differential Gene Expression Analysis

**Figure 1:**
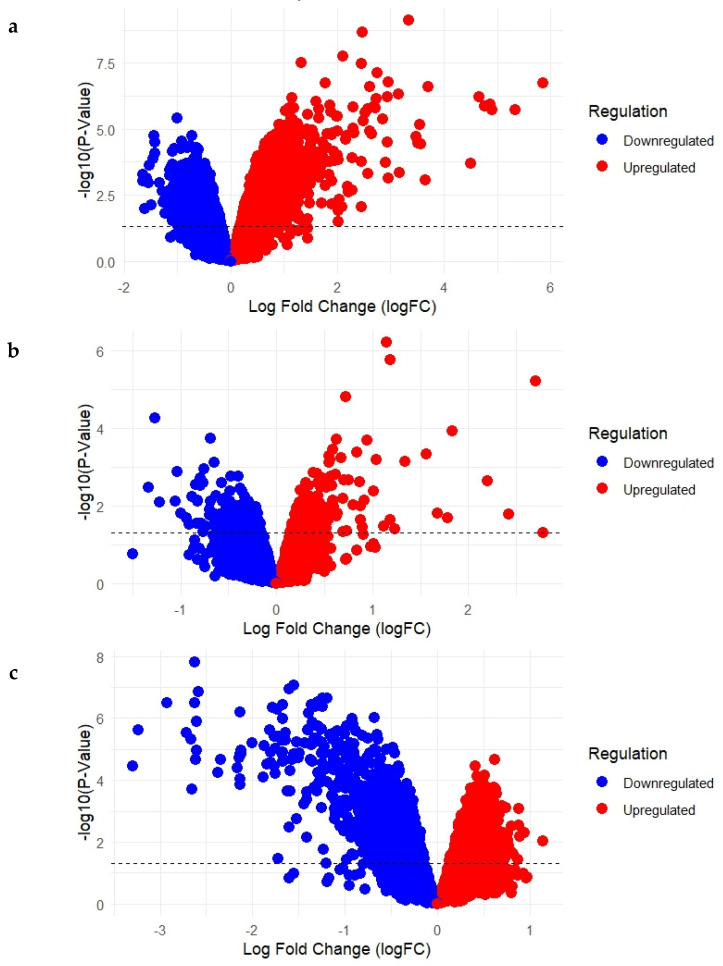
(**a**–**c**): Volcano Plots Showing Upregulated and Downregulated Genes in Acne Lesions and Following Short- and Long-Term Isotretinoin Treatment. (**a**) GSE6475: Acne lesions vs. normal skin; (**b**) GSE10433: Baseline vs. 1-week isotretinoin treatment; (**c**) GSE11792: Baseline vs. 8-week isotretinoin treatment).

Differential gene expression analysis was conducted for each of the three selected datasets to identify genes that were significantly upregulated or downregulated in Acne Vulgaris and following isotretinoin treatment. The outcomes are summarized in the volcano plots presented in Figure 1a–c.

In GSE6475 (Figure 1a), which compared acne lesion samples with normal skin samples, a total of 22,277 differentially expressed genes were identified. Of these, 9570 genes were upregulated and 12,707 were downregulated. These findings represent the baseline molecular alterations associated with acne lesions relative to healthy skin. In GSE10433 (Figure 1b), which assessed gene expression changes following 1 week of isotretinoin treatment, a total of 22,277 genes were differentially expressed. Among them, 12,105 genes were upregulated, while 10,172 genes were downregulated, suggesting substantial early transcriptional responses to the drug. In GSE11792 (Figure 1c), which evaluated expression profiles after 8 weeks of isotretinoin treatment, 22,277 genes were differentially regulated, with 11,844 genes upregulated and 10,433 genes downregulated. This dataset reflects the long-term transcriptional adaptations induced by isotretinoin treatment.

### 2.2. Meta-Analysis

Among the differentially expressed genes identified across the three datasets, several were associated with pathways involved in inflammation and immune regulation. From this group, SPTBN1 and SIPA1L1 were selected for focused analysis based on their consistent regulation patterns in response to isotretinoin treatment and their biological relevance to skin barrier integrity and immune signaling. Both genes demonstrated significant dysregulation in acne lesions and showed recovery toward baseline levels following treatment, indicating their potential roles in mediating the anti-inflammatory effects of isotretinoin.

#### 2.2.1. SPTBN1 (Spectrin Beta, Non-Erythrocytic 1)

**Figure 2:**
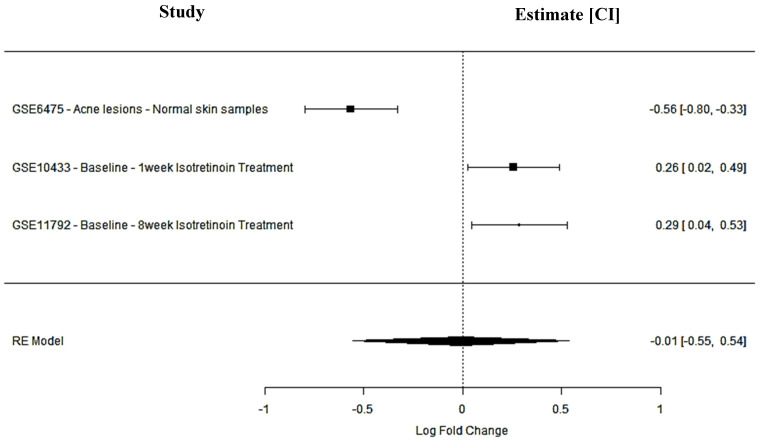
SPTBN1 meta-analysis findings across studies.

SPTBN1 encodes a cytoskeletal protein involved in cellular adhesion and membrane stability—key processes in maintaining skin barrier integrity and modulating inflammation. In acne lesions, SPTBN1 was significantly downregulated (GSE6475: logFC = −0.56; 95% CI: −0.80 to −0.33), suggesting a potential loss of structural regulation contributing to inflammatory pathogenesis. Following isotretinoin treatment, expression was upregulated both after 1 week (GSE10433: logFC = 0.26; 95% CI: 0.02 to 0.49) and 8 weeks (GSE11792: logFC = 0.29; 95% CI: 0.04 to 0.53). However, the random-effects model estimated a non-significant pooled effect size (logFC = −0.01; 95% CI: −0.55 to 0.54; *p* = 0.98), with high heterogeneity (*I*^2^ = 93.8%). Despite statistical variability, the consistent direction of upregulation post-treatment suggests that agonists of SPTBN1 may support cytoskeletal reorganization and barrier restoration, offering a potential anti-inflammatory strategy in acne drug development (Figure 2).

#### 2.2.2. SIPA1L1 (Signal-Induced Proliferation-Associated 1 Like 1)

**Figure 3:**
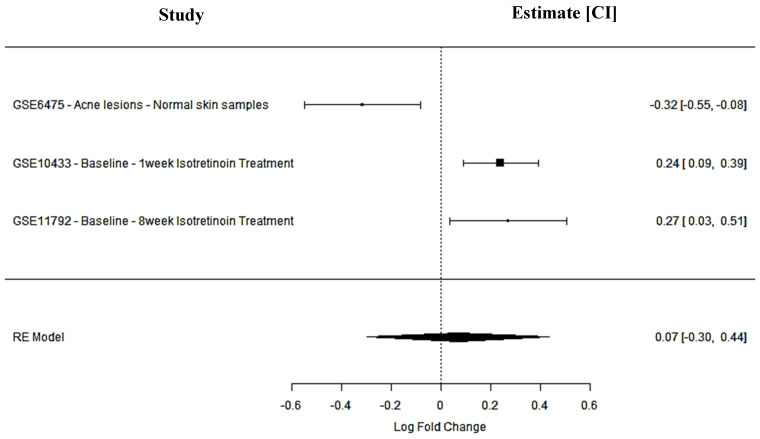
SIPA1L1 meta-analysis findings across studies.

SIPA1L1 is involved in signal transduction pathways critical to immune regulation and cell adhesion. It was downregulated in acne lesions (GSE6475: logFC = −0.32; 95% CI: −0.55 to −0.08), indicating a suppression of immune signaling pathways. Post-isotretinoin treatment, SIPA1L1 was significantly upregulated in both short-term (GSE10433: logFC = 0.24; 95% CI: 0.09 to 0.39) and long-term (GSE11792: logFC = 0.27; 95% CI: 0.03 to 0.51) datasets. The pooled meta-analysis revealed a positive but non-significant effect (logFC = 0.07; 95% CI: −0.30 to 0.44; *p* = 0.71; *I*^2^ = 89.7%), reflecting inter-study variability. Nevertheless, the treatment-induced expression pattern supports SIPA1L1 as a candidate agonist target to enhance immune regulation and attenuate inflammation in acne-prone skin (Figure 3, Table 1).

### 2.3. Structural Elucidation of SPTBN1 and SIPA1L1

**Figure 4:**
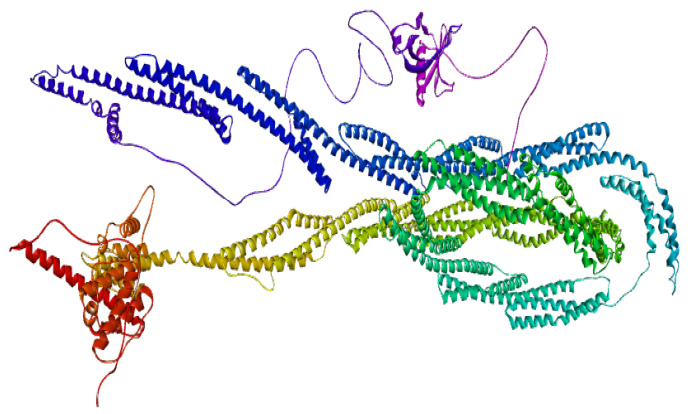
3D structure of SIPA1L1.

SIPA1L1 (Signal-Induced Proliferation-Associated 1-Like Protein 1), encoded by the UniProt entry O43166, is a large human protein comprising 1803 amino acids, structurally characterized by multiple functional domains and regulatory features. It contains a Rap-GAP domain (residues 613–830), responsible for GTPase-activating activity toward Rap1, and a PDZ domain (residues 967–1045), which facilitates protein–protein interactions within signaling complexes and cytoskeletal networks. Several intrinsically disordered regions are distributed throughout the sequence (notably residues 1–157, 1067–1128, 1144–1207, and others), supporting the protein’s role in dynamic cellular signaling. A coiled-coil region near the C-terminus (residues 1735–1795) may assist in protein oligomerization. SIPA1L1 also features multiple compositional bias regions, enriched in polar or charged residues, contributing to its interaction potential. Importantly, the protein is extensively phosphorylated at serine and threonine residues, including modifications by kinases such as PLK2 and CDK5, which may regulate its subcellular localization and activity. Variants such as a somatic mutation at residue 996 and a known SNP (rs12884638) at residue 56 have been reported. This structural profile highlights SIPA1L1 as a highly regulated scaffold protein, and its inducible expression following isotretinoin treatment underscores its therapeutic potential. The presence of accessible domains and regulatory hotspots makes it an attractive candidate for agonist-based drug discovery in the context of immune modulation and Acne Vulgaris treatment (Figure 4).

SPTBN1 (Spectrin Beta, Non-Erythrocytic 1), encoded by the UniProt entry Q01082, is a large cytoskeletal scaffolding protein comprising 2362 amino acids. It plays a critical role in maintaining membrane stability, cellular architecture, and signaling by anchoring membrane proteins to the actin cytoskeleton. The N-terminal region contains two calponin-homology (CH) domains (residues 54–158 and 173–278), which contribute to actin-binding capability. This is followed by 17 spectrin repeats—each ~100 amino acids in length—spanning residues 303 to 2097. These repeats form triple-helical coiled-coil structures that provide mechanical elasticity and flexibility to the protein. A pleckstrin homology (PH) domain is present near the C-terminus (residues 2197–2307), which is implicated in membrane association and protein–lipid interactions. Several regions of intrinsic disorder (e.g., residues 2089–2196 and 2309–2364) contribute to conformational plasticity and may regulate interactions with other cytoskeletal or membrane-associated proteins.

**Figure 5:**
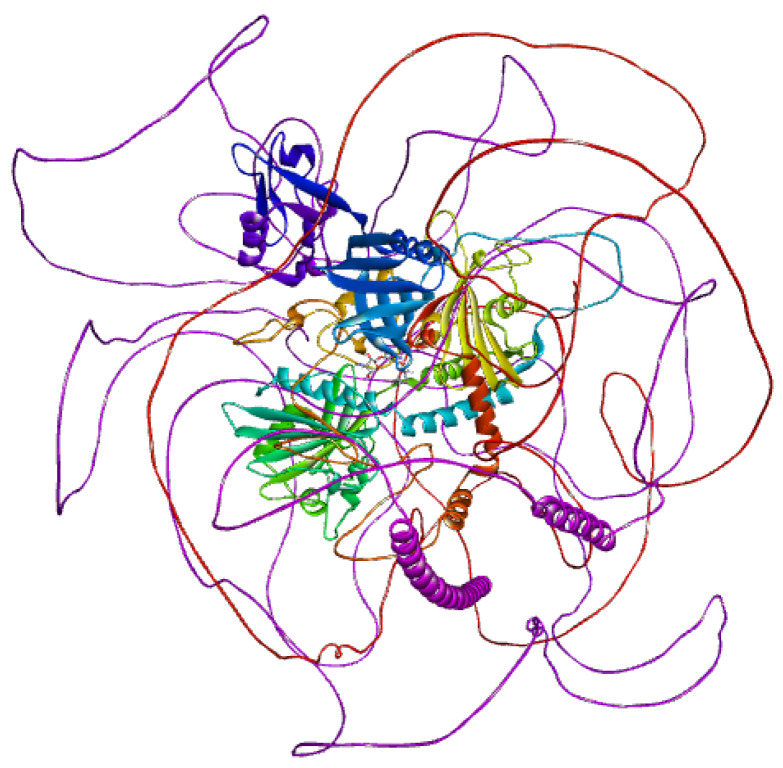
3D structure of SPTBN1.

Functionally relevant interaction domains include a large segment (residues 1563–2093) responsible for binding ankyrin-2 (ANK2), as well as a motif mediating interaction with CAMSAP1 (residues 2149–2177), which links microtubules to the actin cytoskeleton. Numerous post-translational modifications (PTMs) have been identified, especially phosphorylation at serine and threonine residues throughout the spectrin repeats and C-terminal region, which may regulate cytoskeletal dynamics. Acetylation and glycosylation sites have also been observed. Variants affecting cytoskeletal binding and spectrin function have been linked to cytoskeletal dysregulation, particularly in neurons. Given its dynamic regulation and interaction with both membrane and cytoskeletal proteins, SPTBN1 represents a compelling drug target for restoring cytoskeletal integrity and barrier function, particularly in inflammatory skin disorders like Acne Vulgaris (Figure 5).

### 2.4. Molecular Docking Results

To explore the therapeutic potential of selected small molecules in modulating SIPA1L1 and SPTBN1 activity, molecular docking was performed against the protein structures corresponding to SIPA1L1 (UniProt ID: O43166) and SPTBN1 (UniProt ID: Q01082). The binding affinities of each compound were evaluated and are presented in Table 2.

Among the tested compounds, Gentamicin (PubChem ID: 3467) exhibited the strongest binding affinity to both targets, with a binding score of −8.6 kcal/mol against SIPA1L1 and −5.9 kcal/mol against SPTBN1, indicating high binding potential and broad target engagement. In contrast, the other naturally derived compounds showed moderate to weak binding interactions.

Citral (PubChem ID: 638011) and Geraniol (PubChem ID: 637566) showed comparable affinities toward SIPA1L1 (−5.3 and −5.1 kcal/mol, respectively), while Citronellal (PubChem ID: 7794) and Citronellol (PubChem ID: 8842) had slightly lower binding scores (−5.1 and −4.9 kcal/mol). Binding affinities to SPTBN1 were generally lower across all natural compounds, ranging from −4.4 to −3.9 kcal/mol, suggesting relatively weaker interactions with this protein target.

Overall, these findings highlight Gentamicin as the most promising candidate for further in vitro and in silico validation targeting both inflammation-related proteins. Natural compounds such as Citral and Geraniol may serve as mild modulators of SIPA1L1 activity, warranting additional exploration in functional assays.

#### 2.4.1. Binding Pocket Interactions of Gentamicin with SPTBN1

**Figure 6:**
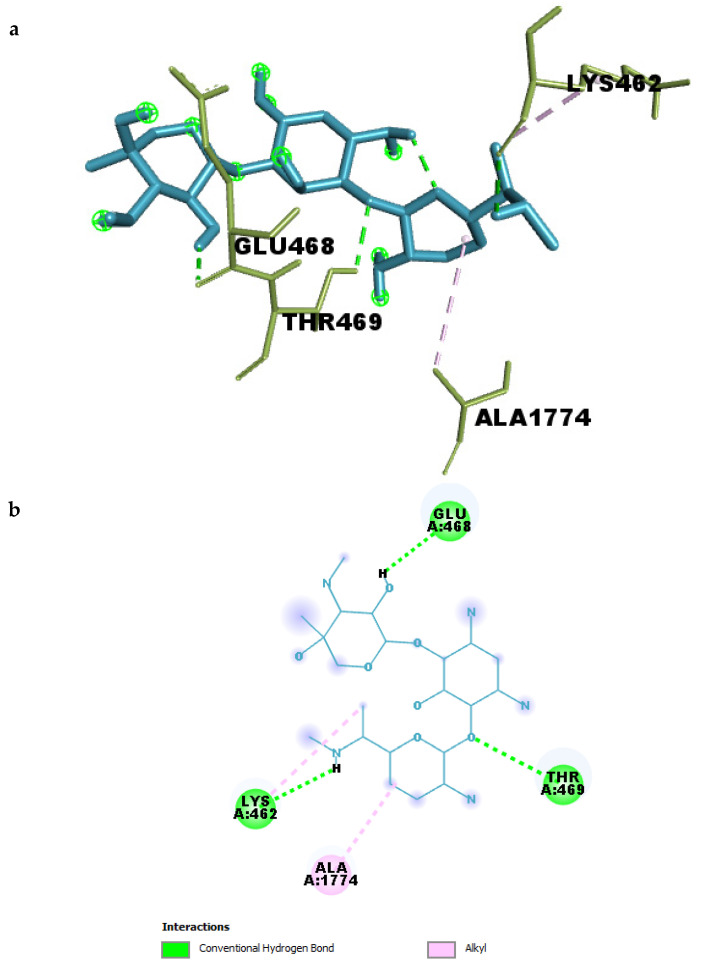
(**a**) 3D and (**b**) 2D interaction of Gentamicin within the binding pocket of SPTBN1.

To further understand the molecular basis of Gentamicin’s binding to SPTBN1, the compound–target complex was analyzed to identify key residue-level interactions within the binding pocket. As summarized in Table 3 (Figure 6a,b), Gentamicin formed multiple conventional hydrogen bonds and hydrophobic contacts that contribute to the stability of the complex.

Four strong hydrogen bonds were observed between Gentamicin and residues THR469, GLU468, LYS462, and an internal ligand oxygen atom. The hydrogen bond between Gentamicin and GLU468 had the shortest distance (2.19 Å), indicating a particularly strong interaction that likely anchors the ligand in the binding site. Similarly, interactions with THR469 and LYS462 had bond lengths of 2.49 Å and 2.59 Å, respectively, suggesting additional stabilization through polar interactions.

In addition to hydrogen bonding, hydrophobic interactions were identified between Gentamicin and ALA1774 and LYS462, both categorized as alkyl interactions, with distances ranging from 3.93 Å to 4.68 Å. These non-polar contacts may assist in optimizing ligand orientation within the pocket and contribute to the overall binding affinity.

#### 2.4.2. Molecular Dynamics Simulation of Gentamicin–SPTBN1 Complex

**Figure 7:**
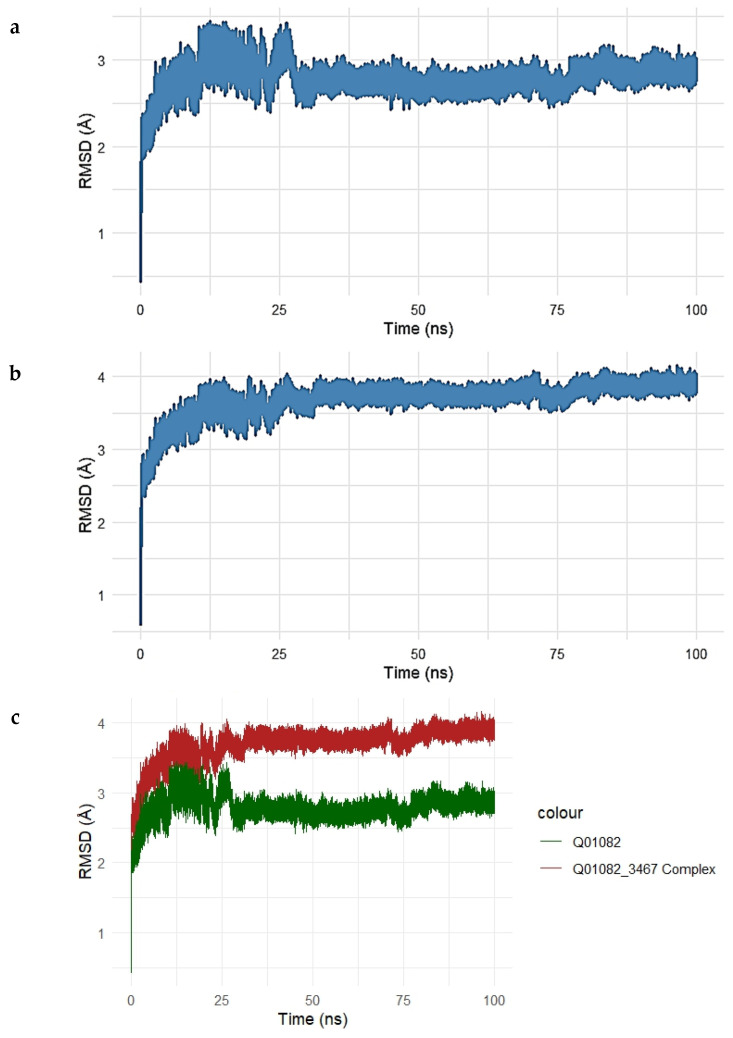
(**a**–**c**). Root Mean Square Deviation (RMSD) analysis of the SPTBN1–Gentamicin complex over a 100 ns molecular dynamics simulation. (**a**) RMSD trajectory of the unbound SPTBN1 protein backbone shows initial structural adjustments stabilizing between 2.5 and 3.2 Å. (**b**) RMSD of the Gentamicin-bound SPTBN1 complex demonstrates greater deviations, stabilizing around 3.5–4.1 Å, indicative of ligand-induced conformational changes. (**c**) Overlay of RMSD profiles for the unbound protein (green) and the complex (red) highlights increased deviation upon Gentamicin binding, suggesting the adoption of a new stable conformation driven by protein–ligand interactions.

To assess the dynamic stability and conformational behavior of the Gentamicin–SPTBN1 complex, a 100 ns molecular dynamics simulation was performed. The root mean square deviation (RMSD) of the protein backbone and the protein–ligand complex was monitored over time to evaluate structural deviations and complex stability, as shown in Figure 7a–c.

The RMSD of the unbound SPTBN1 protein backbone (Figure 7a) showed an initial sharp rise within the first 10 ns, stabilizing around 2.5–3.2 Å for the remainder of the simulation. This indicates that the unbound protein maintained a relatively stable conformation with moderate flexibility.

For the Gentamicin–SPTBN1 complex (Figure 7b), the RMSD increased more significantly, stabilizing at a slightly higher range of 3.5–4.1 Å. This suggests additional conformational adjustments induced by ligand binding, which is expected in response to interactions within the binding pocket. The overlay plot comparing RMSD profiles of the unbound protein and the ligand-bound complex (Figure 7c) clearly illustrates the difference in structural deviation. The complex (red line) exhibits higher RMSD values compared to the unbound protein (green line), indicating that Gentamicin binding introduces conformational changes in SPTBN1, potentially stabilizing a new functional conformation.

Overall, the MD simulation confirms that the Gentamicin–SPTBN1 complex is dynamically stable over the 100 ns simulation window, with RMSD values plateauing after initial equilibration. These results support the binding interactions observed in docking studies and further validate the potential of Gentamicin as a modulator of SPTBN1.

##### Energy Stability of the Gentamicin–SPTBN1 Complex

**Figure 8:**
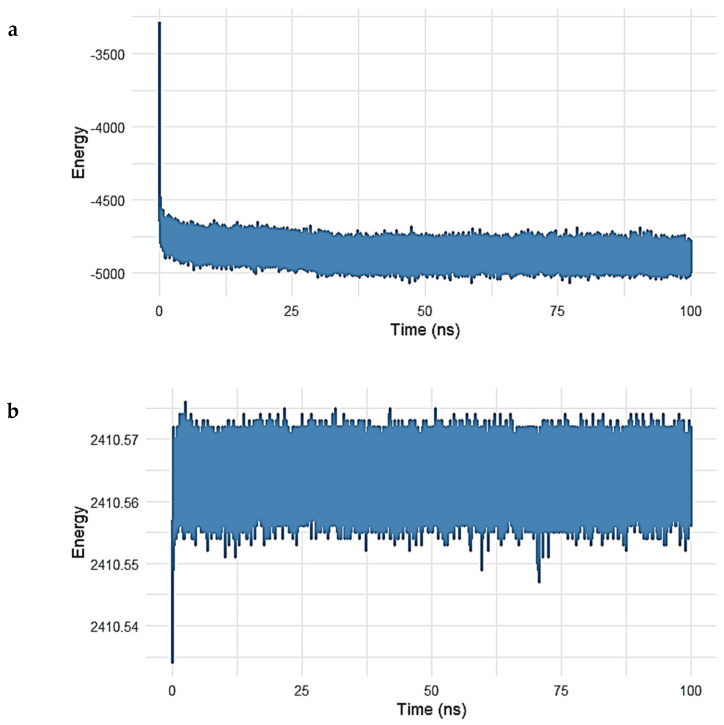
(**a**,**b**). Energy profile of the Gentamicin–SPTBN1 complex during 100 ns molecular dynamics simulation. (**a**) Potential energy trajectory shows a rapid decrease during the initial 5 ns, followed by stabilization around −5000 kcal/mol, indicating system equilibration and thermodynamic stability. (**b**) Kinetic energy remains consistently stable near 2410.56 kcal/mol throughout the simulation, reflecting minimal fluctuations in atomic motion and temperature. Together, these energy profiles confirm the energetic stability and reliability of the Gentamicin–SPTBN1 binding conformation.

The energetic stability of the Gentamicin–SPTBN1 complex was further evaluated by monitoring the potential and kinetic energy throughout the 100 ns molecular dynamics simulation. The energy profiles are presented in Figure 8a,b.

As shown in Figure 8a, the potential energy of the system rapidly decreased within the initial 5 ns, stabilizing around −5000 kcal/mol for the remainder of the simulation. This early decline reflects system equilibration, while the stable energy plateau thereafter indicates that the complex reached a thermodynamically favorable state, maintaining consistent internal energy over time.

Similarly, the kinetic energy of the system (Figure 8b) remained remarkably constant after the initial adjustment phase. Fluctuations were minimal, with values centered around 2410.56 kcal/mol, suggesting that temperature and atomic motion were stable during the simulation period.

The combination of stable potential and kinetic energy profiles confirms that the Gentamicin–SPTBN1 complex remained energetically stable under simulated physiological conditions. These findings support the reliability of the binding conformation observed during docking and MD analysis, reinforcing the candidacy of Gentamicin as a potential modulator of SPTBN1 activity.

##### Interaction Energy Decomposition Analysis of the Gentamicin–SPTBN1 Complex

**Figure 9:**
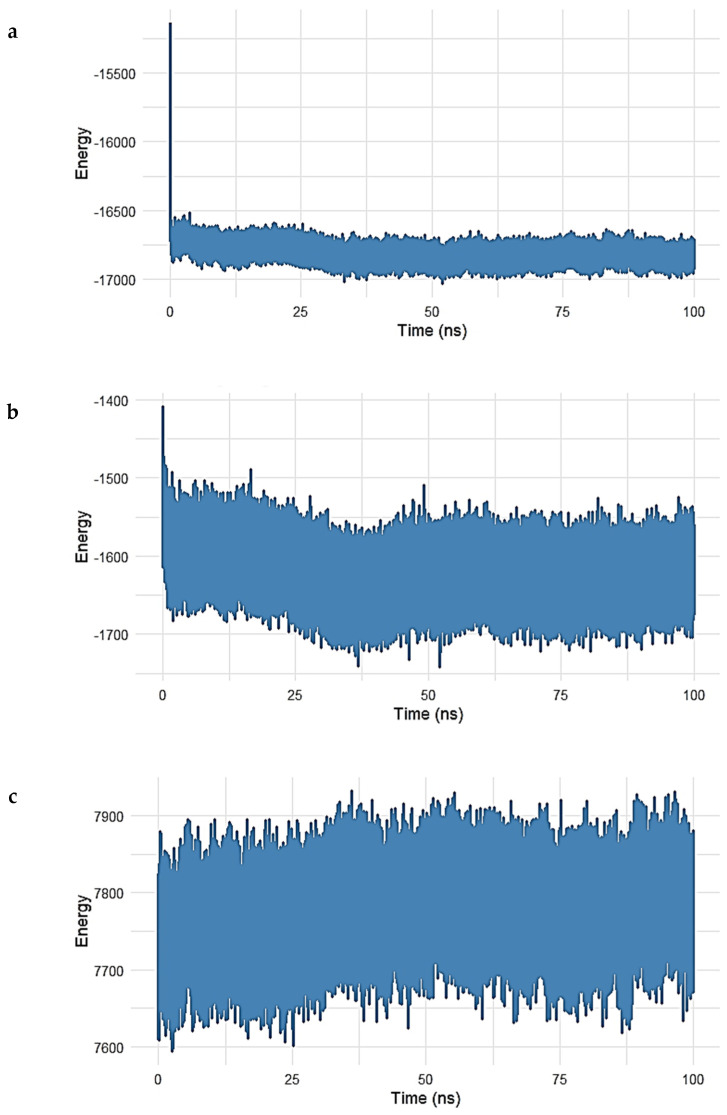

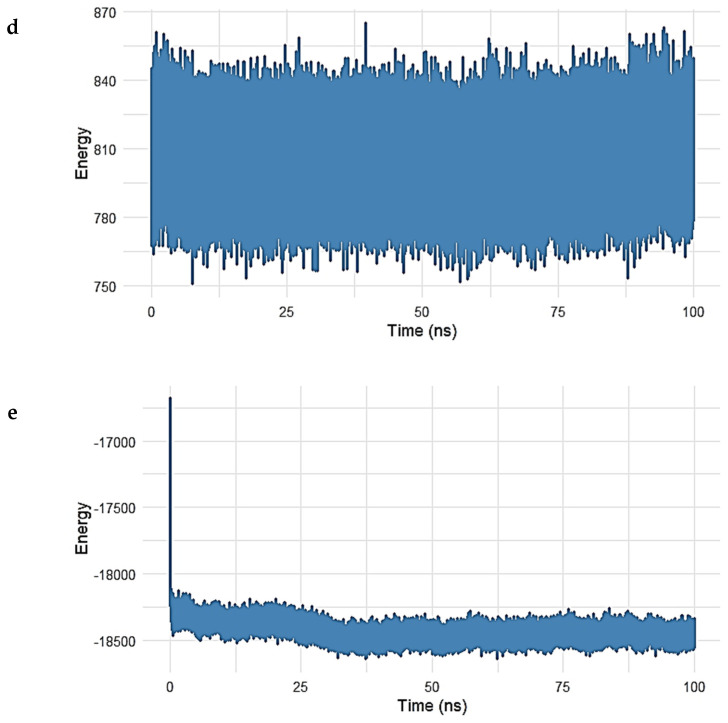
(**a**–**e**). Interaction energy decomposition of the Gentamicin–SPTBN1 complex over a 100 ns molecular dynamics simulation. (**a**) Long-range electrostatic energy stabilized around −17,000 kcal/mol, indicating strong electrostatic interactions. (**b**) Long-range van der Waals energy plateaued near −1650 kcal/mol, reflecting stable non-polar interactions. (**c**) Electrostatic 1–4 interactions and (**d**) vdW 1–4 interactions exhibited consistent values around 7800 kcal/mol and 830 kcal/mol, respectively, confirming intramolecular stability. (**e**) The total interaction energy, combining vdW and electrostatic terms, remained stable near −18,500 kcal/mol, supporting a robust and energetically favorable binding of Gentamicin within the SPTBN1 pocket.

To gain deeper insight into the energetic contributions governing the stability of the Gentamicin–SPTBN1 complex, a decomposition analysis of molecular mechanics energy terms was performed over a 100 ns molecular dynamics simulation. The results are shown in Figure 9a–e.

In Figure 9a, the long-range electrostatic energy exhibited a steady and favorable profile throughout the simulation, stabilizing around −17,000 kcal/mol after an initial drop during equilibration. This suggests strong electrostatic attraction between Gentamicin and the surrounding protein environment, which plays a key role in ligand binding. Figure 9b shows the long-range van der Waals (vdW) energy, which stabilized near −1650 kcal/mol, confirming the presence of persistent non-polar interactions between the ligand and hydrophobic residues in the binding pocket. While exhibiting slightly more fluctuation than electrostatic terms, these vdW contributions remained negative and steady, indicative of stable dispersion interactions.

**Figure 10:**
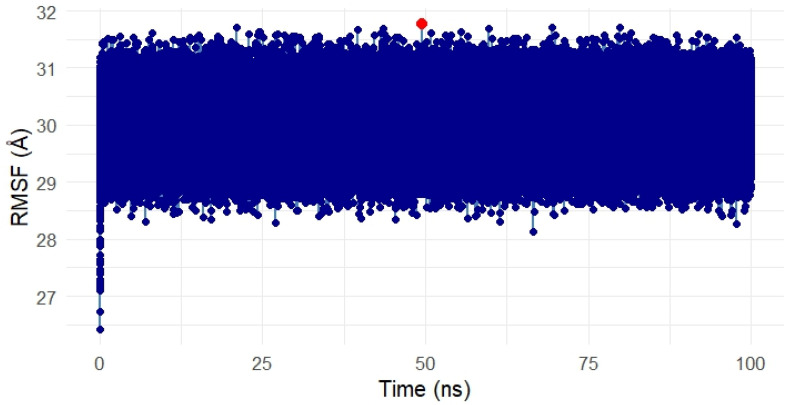
Root Mean Square Fluctuation (RMSF) analysis of the Gentamicin–SPTBN1 complex over 100 ns molecular dynamics simulation. The majority of residues exhibited minimal fluctuations between 28 Å and 32 Å, indicating a stable and rigid protein structure. A single localized spike (in red) reflects slight flexibility around a surface-exposed region but does not affect overall complex stability. The RMSF results confirm that Gentamicin binding does not induce significant structural disruption in SPTBN1.

In the 1–4 interaction analysis, which considers bonded atoms separated by three covalent bonds, both electrostatic 1–4 interactions (Figure 9c) and vdW 1–4 interactions (Figure 10) maintained narrow fluctuation ranges, centered around 7800 kcal/mol and 830 kcal/mol, respectively. These stable patterns confirm the structural integrity and proper conformational behavior of the bonded interaction network during simulation.

Finally, Figure 9e shows the total interaction energy (sum of long-range vdW and electrostatic contributions). The curve stabilized around −18,500 kcal/mol, supporting the conclusion that Gentamicin maintained a strong and energetically favorable binding mode within the SPTBN1 binding pocket across the simulation timeframe.

Collectively, the energy decomposition analysis reveals that both electrostatic and van der Waals forces significantly contributed to the stability of the complex, reinforcing docking and RMSD results and highlighting Gentamicin’s potential as a viable therapeutic lead for targeting SPTBN1.

##### Residue Flexibility Analysis of the Gentamicin–SPTBN1 Complex

To assess the residue-level flexibility and dynamic behavior of the SPTBN1 (Q01082) protein in complex with Gentamicin, a Root Mean Square Fluctuation (RMSF) analysis was conducted over the 100 ns simulation period. The resulting RMSF plot is presented in Figure 10.

Throughout the simulation, the RMSF values remained tightly clustered, with the majority of residues fluctuating within a narrow range between 28 Å and 32 Å, indicating a relatively rigid protein structure with limited local flexibility. This overall low fluctuation suggests that the protein maintained a stable conformation in the presence of the ligand.

A single residue spike, marked in red on the plot, indicates a region with slightly elevated flexibility around the 50 ns mark. However, the deviation was marginal and did not impact the global structural stability of the complex. Such localized motion may reflect a surface loop or a solvent-exposed residue adapting to ligand binding but is not indicative of overall instability.

These RMSF findings are consistent with RMSD and energy analyses, further confirming that Gentamicin binding does not induce significant structural perturbation and that the complex remains conformationally stable during the simulation.

### 2.5. Binding Pocket Interactions of Gentamicin with SIPA1L1

**Figure 11:**
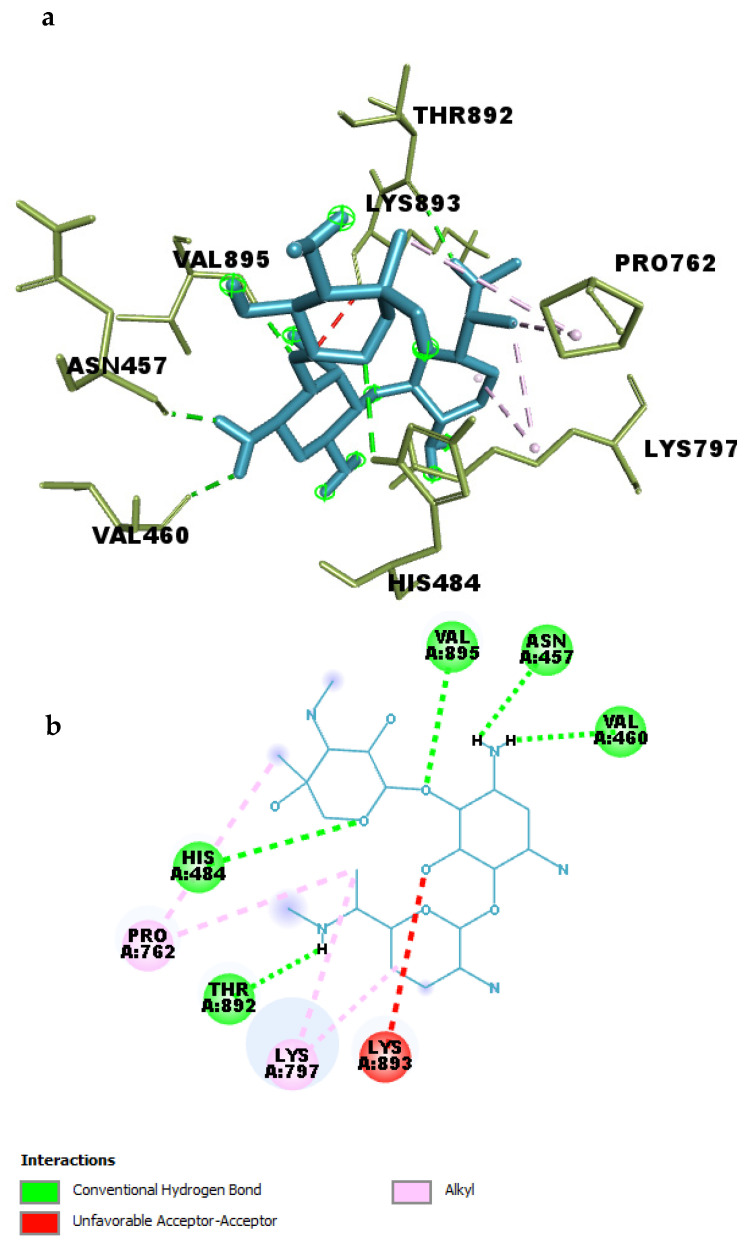
(**a**) 3D and (**b**) 2D interaction of Gentamicin within the binding pocket of SIPA1L1.

Molecular docking analysis revealed that Gentamicin formed a stable and well-oriented complex within the binding pocket of SIPA1L1, engaging in multiple hydrogen bonds and hydrophobic interactions, as detailed in Table 4 (Figure 11a,b).

A total of five conventional hydrogen bonds were identified between Gentamicin and key residues within the binding site. Notably, a strong hydrogen bond was formed with ASN457 at a distance of 2.05 Å, indicating a highly favorable polar interaction. Additional hydrogen bonds were observed with VAL895 (2.29 Å), THR892 (2.15 Å), VAL460 (2.28 Å), and HIS484 (2.93 Å), highlighting multiple anchoring points that may enhance the specificity and strength of binding.

In addition to polar contacts, four alkyl-based hydrophobic interactions were detected between Gentamicin and residues LYS797 and PRO762, with distances ranging from 3.77 Å to 5.30 Å. These interactions likely contribute to the optimal positioning of the ligand within the hydrophobic environment of the pocket and help stabilize the complex.

Together, these interactions explain the high binding affinity of Gentamicin toward SIPA1L1 observed during docking (−8.6 kcal/mol). The interaction profile suggests that Gentamicin engages in both strong electrostatic and hydrophobic contacts, supporting its potential as a lead compound for modulating SIPA1L1 in inflammation-related pathways.

#### 2.5.1. Molecular Dynamics Simulation of the Gentamicin–SIPA1L1 Complex

**Figure 12:**
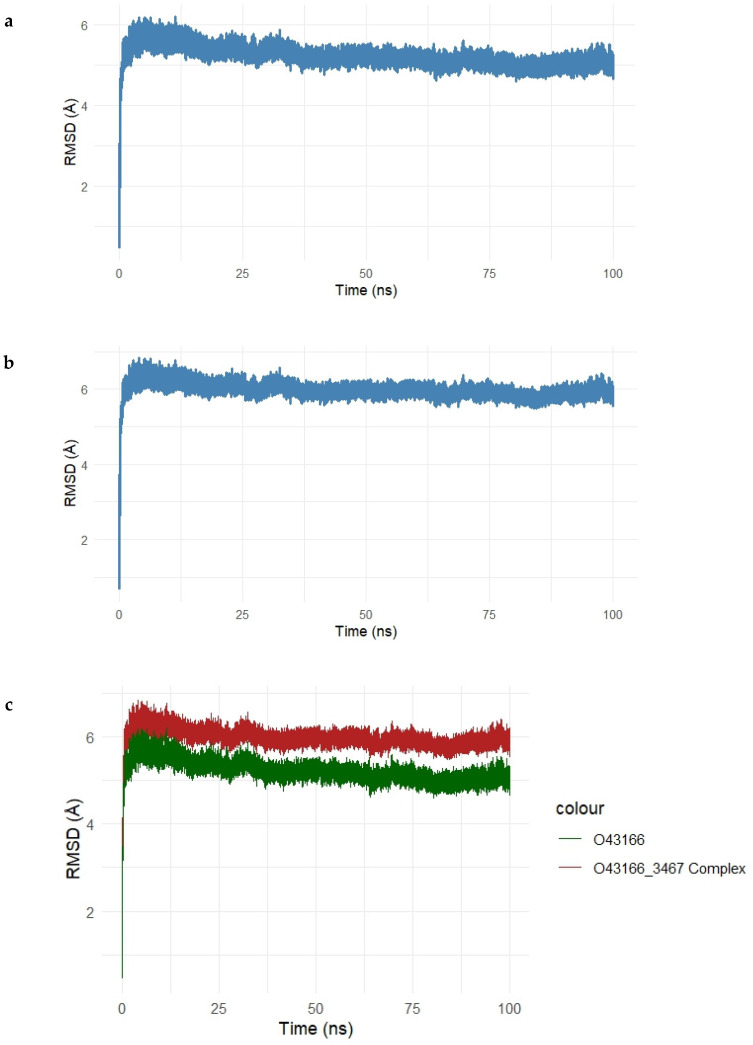
(**a**–**c**). Root Mean Square Deviation (RMSD) analysis of the Gentamicin–SIPA1L1 complex during 100 ns molecular dynamics simulation. (**a**) RMSD of the unbound SIPA1L1 protein stabilizes between 5.5 Å and 6.2 Å, indicating structural equilibrium. (**b**) The Gentamicin-bound complex exhibits slightly higher RMSD values, ranging from 6.0 Å to 6.5 Å, reflecting mild ligand-induced adjustments. (**c**) Overlay of unbound (green) and complexed (red) trajectories highlights the increased but stable deviation upon ligand binding, confirming the conformational stability of the complex throughout the simulation.

To evaluate the conformational stability and structural dynamics of the Gentamicin–SIPA1L1 complex, a 100 ns molecular dynamics simulation was conducted. The root mean square deviation (RMSD) of the unbound protein and the protein–ligand complex was tracked over time to assess stability and ligand-induced conformational changes, as shown in Figure 12a–c.

In Figure 12a, the RMSD of the unbound SIPA1L1 protein backbone showed an initial rise within the first 10 ns and plateaued between 5.5 Å and 6.2 Å, indicating that the protein reached equilibrium and maintained its structural integrity during the simulation. As shown in Figure 12b, the Gentamicin–SIPA1L1 complex exhibited a similar trend, with RMSD values stabilizing slightly above the unbound protein, ranging from 6.0 Å to 6.5 Å. This suggests that while Gentamicin binding introduces mild structural deviations, the complex remains conformationally stable.

The overlay plot (Figure 12c) directly compares the RMSD trajectories of the unbound protein (green) and the protein–ligand complex (red). The complex consistently maintained higher RMSD values than the unbound form, indicating ligand-induced conformational adjustments, yet with minimal fluctuation beyond 6.5 Å, confirming the overall dynamic stability of the complex.

Collectively, these findings demonstrate that Gentamicin maintains stable binding within the SIPA1L1 binding pocket, supporting the structural compatibility and reinforcing its potential as a candidate for therapeutic modulation.

##### Energy Stability Analysis of the Gentamicin–SIPA1L1 Complex

**Figure 13:**
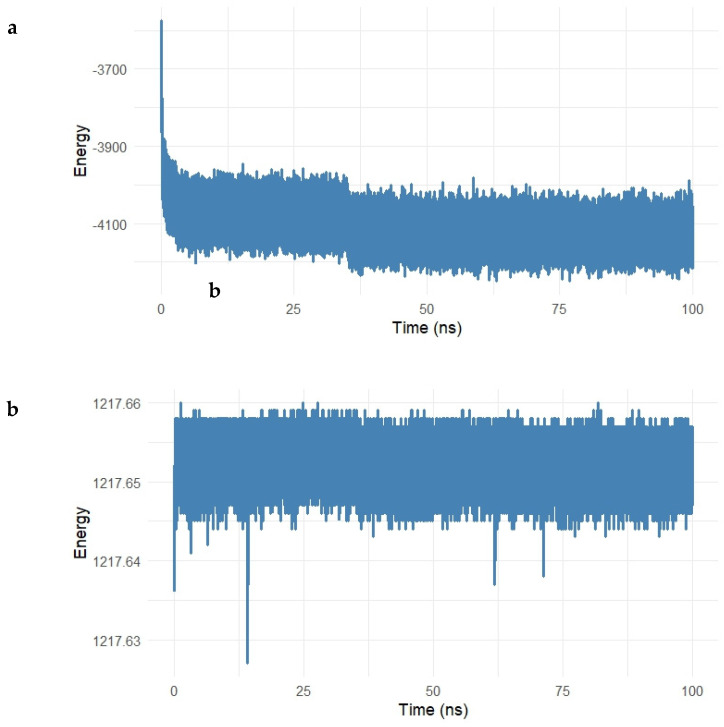
(**a**,**b**). Potential and kinetic energy profiles of the Gentamicin–SIPA1L1 complex over a 100 ns molecular dynamics simulation. (**a**) The potential energy rapidly decreased during the initial 10 ns and stabilized around −4100 kcal/mol, indicating system equilibration and a thermodynamically favorable binding state. (**b**) Kinetic energy remained stable around 1217.65 kcal/mol with minimal fluctuations, reflecting consistent temperature control and confirming the energetic stability of the complex under simulation conditions.

To evaluate the thermodynamic stability of the Gentamicin–SIPA1L1 complex, potential and kinetic energy profiles were analyzed throughout a 100 ns molecular dynamics simulation, as presented in Figure 13a,b.

As shown in Figure 13a, the potential energy of the system decreased rapidly during the first 10 ns, reflecting the system’s equilibration, and subsequently stabilized around −4100 kcal/mol for the remaining simulation period. This steady energy plateau suggests the formation of a thermodynamically favorable complex, with no major energetic disruptions occurring throughout the simulation.

The kinetic energy profile (Figure 13b) remained nearly constant during the simulation, with values fluctuating minimally around 1217.65 kcal/mol. The low level of fluctuation indicates consistent temperature and energy distribution within the system, further confirming that the dynamics of the Gentamicin–SIPA1L1 complex are stable under physiological simulation conditions.

##### Interaction Energy Decomposition Analysis of the Gentamicin–SIPA1L1 Complex

**Figure 14:**
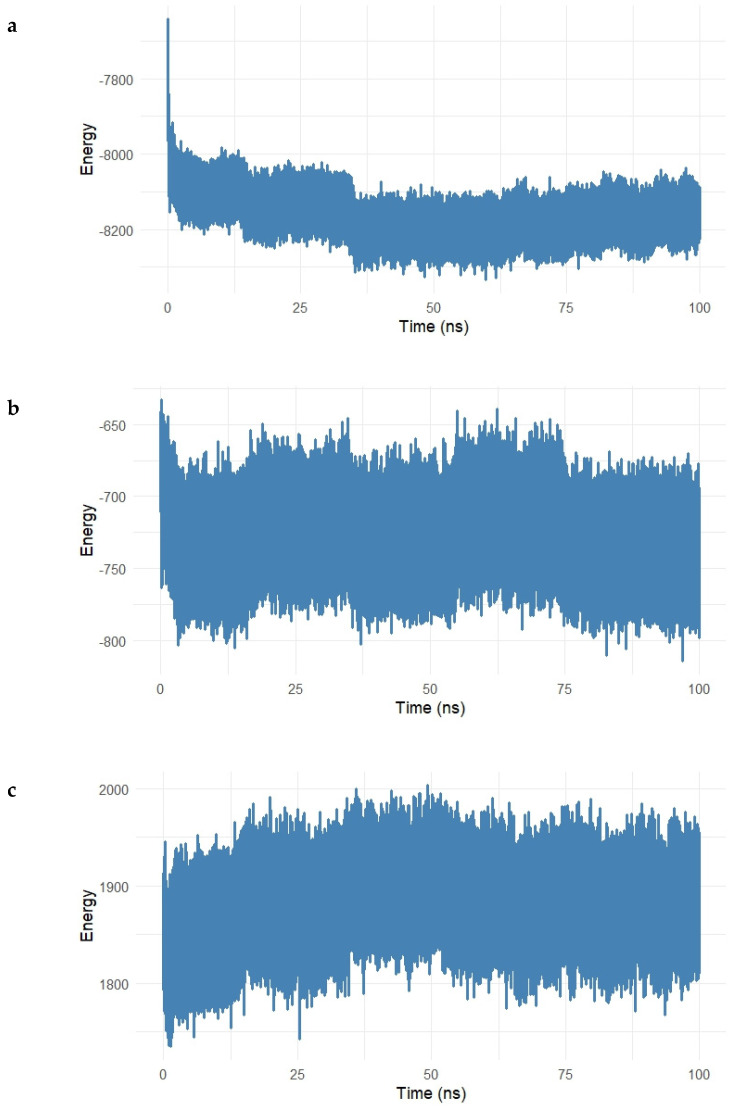

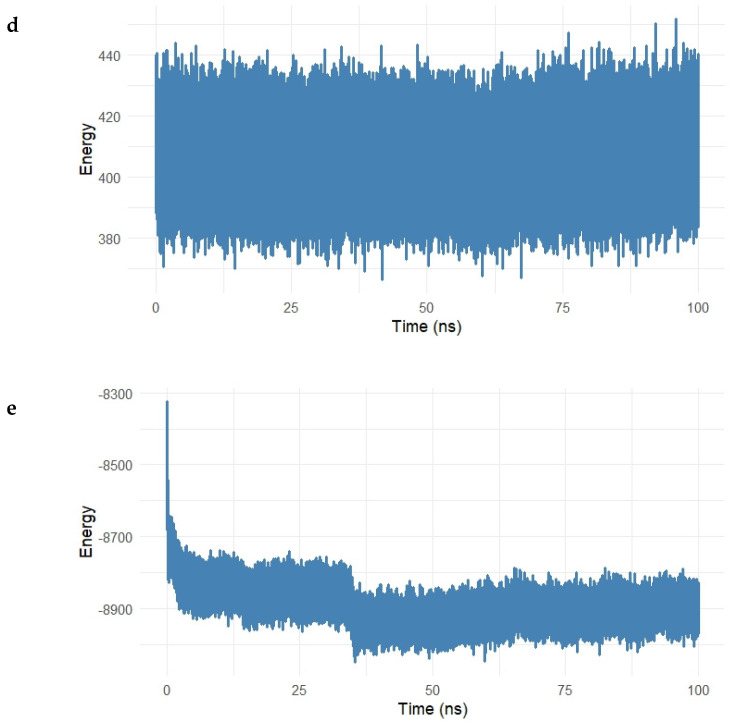
(**a**–**e**). Energy decomposition analysis of the Gentamicin–SIPA1L1 complex over a 100 ns molecular dynamics simulation. (**a**) Long-range electrostatic interactions stabilized around −8200 kcal/mol, indicating strong electrostatic attraction between Gentamicin and SIPA1L1. (**b**) Long-range van der Waals energy fluctuated around −700 kcal/mol, reflecting stable hydrophobic interactions. (**c**,**d**) Electrostatic and van der Waals 1–4 interactions remained centered near 1900 kcal/mol and 420 kcal/mol, respectively, suggesting preserved intramolecular stability. (**e**) Total interaction energy stabilized near −8900 kcal/mol, confirming the formation of a robust and energetically favorable complex.

To further characterize the molecular stability and nature of the Gentamicin–SIPA1L1 (O43166) complex, an energy decomposition analysis was carried out, evaluating the long-range and 1–4 nonbonded interaction energies throughout the 100 ns molecular dynamics simulation. The results are illustrated in Figure 14a–e.

Figure 14a shows the electrostatic long-range interactions, which stabilized around −8200 kcal/mol, following an initial drop during equilibration. This steady negative energy profile reflects persistent electrostatic attraction between Gentamicin and charged residues within the SIPA1L1 binding cavity.

The van der Waals (vdW) long-range energy (Figure 14b) fluctuated around −700 kcal/mol, with typical dynamic noise expected from hydrophobic interactions. Despite some fluctuations, the energy remained consistently negative, indicating stable hydrophobic contacts over the simulation.

**Figure 15:**
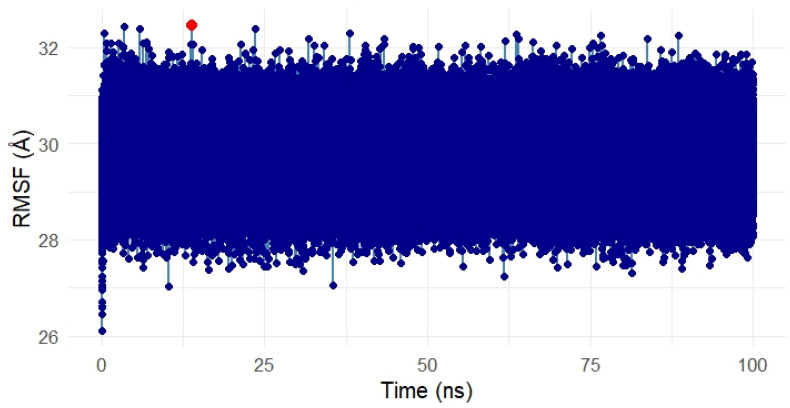
Root Mean Square Fluctuation (RMSF) analysis of the Gentamicin–SIPA1L1 complex over a 100 ns molecular dynamics simulation. The majority of residues showed low fluctuations between ~28 Å and 32 Å, indicating overall conformational stability. Minor localized spikes (highlighted in red) suggest flexible regions likely associated with loop or solvent-exposed areas, while the binding domain remained stable. These results confirm that Gentamicin binding does not destabilize SIPA1L1, supporting its structural robustness as a therapeutic target. In this meta-analysis, SPTBN1 and SIPA1L1 emerged as biologically relevant targets due to their regulatory roles in inflammation, immune signaling, and skin barrier maintenance. Although the pooled expression changes were not statistically significant, likely due to inter-study heterogeneity, both genes displayed consistent treatment-associated recovery trends, suggesting therapeutic relevance. SPTBN1 encodes βII-spectrin, a cytoskeletal protein that maintains epithelial structure and attenuates inflammation by stabilizing SOCS1 and inhibiting NF-κB activation [6,7]. Its downregulation in acne lesions aligns with known cytoskeletal disruptions that contribute to barrier dysfunction and inflammation [8]. SIPA1L1, a Rap GTPase-activating protein, is involved in cytoskeletal remodeling and immune modulation, acting at cell junctions and influencing cellular adhesion and trafficking [9]. Isotretinoin, the most effective systemic anti-acne agent, is known to restore skin barrier homeostasis and reduce pro-inflammatory cytokines [10,11]. Thus, the upregulation of SPTBN1 and SIPA1L1 post-treatment may reflect isotretinoin’s capacity to normalize skin structure and immune balance. These findings, together with docking and MD simulation results, highlight the potential of SPTBN1 and SIPA1L1 as mechanistically aligned therapeutic targets for anti-inflammatory acne interventions.

Short-range interactions were further assessed in Figure 14c and Figure 15 The electrostatic 1–4 interaction energy remained centered around 1900 kcal/mol, while vdW 1–4 interactions fluctuated near 420 kcal/mol, both exhibiting tight ranges, which suggests that the bonded and angular network within the protein–ligand complex remained structurally intact.

The total interaction energy (sum of long-range electrostatic and vdW energies) is depicted in Figure 14e. After an initial decline, the interaction energy stabilized at approximately −8900 kcal/mol, supporting the conclusion that the Gentamicin–SIPA1L1 complex achieves a strong and energetically favorable binding state with no major destabilizing fluctuations.

Altogether, this decomposition analysis reinforces the notion that Gentamicin exhibits consistent and stable interactions with SIPA1L1, both electrostatically and hydrophobically, thus supporting its potential as a candidate for drug repurposing in the treatment of inflammation-associated acne pathogenesis.

##### Flexibility Analysis of the Gentamicin–SIPA1L1 Complex

The Root Mean Square Fluctuation (RMSF) analysis was conducted to assess the residue-level flexibility of the SIPA1L1 protein (UniProt ID: O43166) in complex with Gentamicin over the 100 ns simulation period. Figure 15 presents the RMSF values, reflecting the average positional deviation of each residue throughout the trajectory.

Overall, the RMSF profile remained uniformly compact, with fluctuations primarily ranging between ~28 Å and 32 Å, indicating a low degree of flexibility and structural stability across the protein during ligand binding. A few residues exhibited slightly elevated fluctuations (highlighted in red), suggesting localized motions, potentially around loop regions or surface-exposed segments. However, no drastic fluctuations were observed within the binding domain, suggesting that the ligand-protein interface remained rigid and stable.

These findings confirm that Gentamicin binding to SIPA1L1 does not induce destabilizing conformational shifts, further validating the robustness and rigidity of the interaction at the molecular level and supporting SIPA1L1 as a promising stable therapeutic target for future anti-inflammatory interventions.

Gentamicin emerged as a compelling lead candidate in our study, demonstrating robust in silico binding to the inflammation-associated targets SPTBN1 and SIPA1L1 (notably, SPTBN1 is a known negative regulator of NF-κB–mediated inflammatory signaling [12]). In molecular docking, gentamicin achieved one of the highest binding affinities to both target proteins. Subsequent molecular dynamics (MD) simulations revealed that the gentamicin–protein complexes remained conformationally stable over time. Throughout a 100 ns MD trajectory, gentamicin maintained key interactions within the binding sites of SPTBN1 and SIPA1L1, exhibiting minimal RMSD fluctuations and no tendency to dissociate—a hallmark of stable ligand engagement in silico [13]. This stable conformational behavior was accompanied by favorable interaction energetics. Collectively, these in silico findings strongly support gentamicin’s potential as a repurposed anti-inflammatory agent. As an FDA-approved antibiotic, gentamicin offers the advantage of a well-characterized safety profile. Interestingly, beyond its antimicrobial action, it can also exert immunomodulatory effects—for example, promoting regulatory T cells and reducing cytokine-mediated tissue damage in vivo [14]. This dual antimicrobial and anti-inflammatory potential makes gentamicin particularly attractive for targeting acne-related inflammatory pathways. Furthermore, our results underscore the value of combining molecular docking with MD simulations in early-stage drug discovery and target validation. The docking step efficiently screens and ranks candidates by predicted binding affinity, while the MD step validates the stability and realistic behavior of the ligand–target complex under dynamic conditions [15]. This integrated approach provides more reliable predictions of therapeutic efficacy in the context of acne inflammation, and it has been shown to expedite the identification of promising leads for drug repurposing [15]. Overall, gentamicin’s strong binding affinity, stable interaction profile, and favorable energetics observed in silico highlight its therapeutic potential against acne-associated inflammation. These findings warrant further experimental validation in relevant biological models.

While gentamicin demonstrated strong binding affinity and conformational stability with both SPTBN1 and SIPA1L1—two inflammation-associated proteins dysregulated in Acne Vulgaris—our docking analysis also revealed that citronella-derived compounds such as citral, citronellol, citronellal, and geraniol displayed moderate but stable interactions with these targets, particularly SIPA1L1. Notably, citral and geraniol formed multiple hydrogen bonds and hydrophobic contacts, suggesting a capacity to modulate immune signaling pathways involved in acne pathogenesis. Although their binding energies were less pronounced than those of gentamicin, these natural compounds nonetheless exhibited interaction profiles indicative of bioactivity, supporting their consideration as adjunctive agents in acne treatment strategies.

Citronella (Cymbopogon winterianus) essential oils are rich in terpenoid compounds known for their antimicrobial, anti-inflammatory, and antioxidant properties [16,17,18]. Citral, for example, has been reported to inhibit NF-κB signaling and reduce pro-inflammatory cytokine production in various models of skin inflammation [19]. Similarly, geraniol has demonstrated immunomodulatory effects, including the suppression of IL-1β and TNF-α expression [20]. These properties align with the functional roles of SIPA1L1 in immune signaling and cell adhesion, suggesting that citronella-derived compounds may contribute to restoring immune balance and barrier integrity in acne-prone skin.

Given their natural origin and well-documented safety profiles, citronella-derived phytochemicals may enhance the therapeutic efficacy of conventional treatments like gentamicin through synergistic anti-inflammatory effects, particularly in formulations designed to target SIPA1L1. The integration of such bioactives may also mitigate the risk of antibiotic resistance by lowering the necessary dosage of antimicrobial agents [21]. Therefore, the observed interaction between citronella-derived compounds and SIPA1L1 supports further investigation into their adjunctive potential, particularly in combinatorial approaches that target both microbial and inflammatory components of acne.

Overall, this study supports a dual-targeting paradigm for acne treatment—addressing both microbial load and inflammatory dysregulation—by combining pharmacological agents like gentamicin with bioactive phytochemicals such as those derived from citronella. Further in vitro and in vivo studies are warranted to validate the mechanistic synergy of such combinations and to assess their clinical relevance in managing chronic acne inflammation.

## 3. Methodology

### 3.1. Data Source and Search Strategy

To identify relevant gene expression studies exploring the effects of isotretinoin in Acne Vulgaris, a systematic search was performed in the NCBI Gene Expression Omnibus (GEO) database. The search strategy employed the following query: (“Acne Vulgaris” [MeSH Terms] OR Acne Vulgaris [All Fields]) AND (“isotretinoin” [MeSH Terms] OR isotretinoin [All Fields]). This search yielded a total of 45 records.

To narrow down the results, only datasets that utilized expression profiling by array as the experimental platform were considered, reducing the number of relevant datasets to 8. These selected datasets were then evaluated based on predefined inclusion and exclusion criteria. Eligible studies were those that involved human samples with a clinical diagnosis of Acne Vulgaris, included gene expression data both before and after isotretinoin treatment, and provided access to either raw or processed expression data. Studies were also required to have clearly defined control and treatment groups with sufficient replicates.

**Figure 16:**
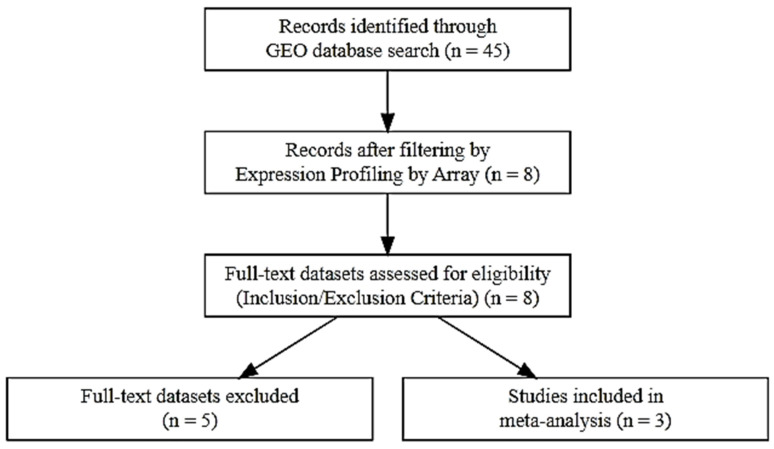
PRISMA flow diagram outlining the study selection process for inclusion in the meta-analysis.

Datasets were excluded if they involved non-human models, lacked isotretinoin intervention, or focused on unrelated dermatological conditions. After applying these criteria, three datasets were deemed suitable and included in the meta-analysis. The overall process of study selection is illustrated in the PRISMA flow diagram presented in Figure 16.

Three gene expression datasets were selected from the GEO database for inclusion in this meta-analysis based on their relevance, experimental design, and adherence to inclusion criteria. These datasets provided valuable insights into the molecular changes associated with Acne Vulgaris and the therapeutic effects of isotretinoin.

The first dataset, **GSE6475**, comprised expression profiles from 18 skin samples, including 6 acne lesion samples, six normal skin samples from acne patients, and six normal skin samples from individuals without acne. This dataset was used to identify baseline gene expression differences associated with acne pathogenesis. The second dataset, **GSE10433**, included a total of 12 samples collected before and after a short-term (1-week) isotretinoin treatment. It consisted of six baseline samples and six samples obtained after treatment, allowing for the identification of early transcriptional responses to isotretinoin therapy. The third dataset, **GSE11792**, involved a more extended treatment duration, with gene expression profiles from 16 samples. These included eight baseline samples and eight samples collected after 8 weeks of isotretinoin administration. This dataset was particularly useful for capturing long-term transcriptional changes induced by isotretinoin (Table 5). Together, these three studies provided a robust basis for examining differentially expressed genes (DEGs) in Acne Vulgaris and understanding the temporal molecular effects of isotretinoin treatment.

### 3.2. Target Protein Retrieval

The target proteins SPTBN1 (Spectrin Beta, Non-Erythrocytic 1; UniProt ID: Q01082) and SIPA1L1 (Signal-Induced Proliferation-Associated 1 Like 1; UniProt ID: O43166) were selected based on transcriptomic meta-analysis that identified their differential expression in acne lesions and response to isotretinoin treatment. Both proteins are associated with inflammation and skin barrier regulation. Since experimentally solved 3D structures were not available in the Protein Data Bank (PDB), high-confidence-predicted structures were obtained from the AlphaFold Protein Structure Database in PDB format for downstream analysis.

### 3.3. Ligand Retrieval

Six ligands were selected based on their documented antimicrobial activity against *Propionibacterium acnes* and other pathogens. These included four plant-derived compounds from *Cymbopogon winterianus* are Geraniol (PubChem ID: 637566), Citral (638011), Citronellal (7794), and Citronellol (8842) and one reference antibiotic, Gentamicin (3467). Ligand structures were retrieved in SDF format from the PubChem database and converted to PDBQT format using Open Babel for compatibility with molecular docking.

### 3.4. Ligand Preparation

Each ligand underwent energy minimization and structural optimization using the MMFF94 force field in PyRx version 0.8 [22]. The optimized 3D conformers were prepared for docking by assigning Gasteiger charges, defining torsions, and converting the structures to PDBQT format using AutoDock Tools version 4.2.6 [23].

### 3.5. Protein Preparation

The AlphaFold-predicted structures of SPTBN1 and SIPA1L1 were prepared for docking by removing water molecules, heteroatoms, and non-protein components. Missing hydrogen atoms were added, and the structures were minimized using the steepest descent algorithm in GROMACS version 5.1.4. The prepared proteins were converted to PDBQT format using AutoDock Tools [24].

### 3.6. Molecular Docking

Molecular docking simulations were carried out using AutoDock Vina to evaluate the binding affinities and interaction profiles between each ligand and the two protein targets. The grid box dimensions were set to encompass the predicted active sites. Binding affinities (in kcal/mol) were recorded and docking poses were visualized in PyMOL version 3.1 and Discovery Studio Visualizer to identify hydrogen bonds, hydrophobic interactions, and other non-covalent interactions within the binding pocket.

### 3.7. Molecular Dynamics Simulation

To assess the structural stability of the top-scoring ligand–protein complexes (Gentamicin–SPTBN1 and Gentamicin–SIPA1L1), 100 ns molecular dynamics (MD) simulations were performed using GROMACS 2021. The AMBER99SB-ILDN force field was used for protein topology, and ligand topologies were generated via the ACPYPE tool. Complexes were solvated in a TIP3P water box with periodic boundary conditions and neutralized by adding counterions. Energy minimization was followed by equilibration in NVT (100 ps) and NPT (100 ps) ensembles before production runs. RMSD, RMSF, potential energy, kinetic energy, and interaction energy decomposition analyses were conducted to evaluate system stability and protein–ligand dynamics.

### 3.8. Post-MD Analysis

The stability and flexibility of the protein–ligand complexes were assessed using root mean square deviation (RMSD), root mean square fluctuation (RMSF), and energy decomposition plots (electrostatic, van der Waals, and total interaction energy). Hydrogen bond analysis and trajectory visualizations were performed using R version 4.5.1.

## 4. Conclusions

This study identified SPTBN1 and SIPA1L1 as inflammation-related targets modulated by isotretinoin. Molecular docking and dynamics confirmed Gentamicin’s strong, stable binding to both proteins. These findings suggest that Gentamicin and *Cymbopogon winterianus*-derived compounds hold promise for anti-inflammatory acne therapy through dual targeting of structural and immune regulatory pathways.

## Figures and Tables

**Table 1:** Inflammation and Immune Response Targets in Acne Vulgaris.

Gene	logFC	SE	*p*-Value	CI_LB	CI_UB	*I* ^2^	tau^2^
SPTBN1	−0.00768	0.278541	0.978007	−0.55361	0.538252	93.82046	0.218364
SIPA1L1	0.068953	0.187613	0.713225	−0.29876	0.436667	89.6881	0.094233

**Table 2:** Binding affinities of Gentamicin and *C. winterianus*-derived compounds against respective targets.

Compounds	PubChem ID	Binding Affinity (kcal/mol)	
		O43166 (SIPA1L1)	Q01082 (SPTBN1)
Gentamicin	3467	−8.6	−5.9
Citronellal	7794	−5.1	−3.9
Citronellol	8842	−4.9	−3.9
Geraniol	637566	−5.1	−4
Citral	638011	−5.3	−4.4

**Table 3:** Chemical interaction based on the interaction of Gentamicin within the binding pocket of SPTBN1.

Name	XYZ: X	XYZ: Y	XYZ: Z	Distance	Category	Types
A:THR469:HG1—N:UNK1:O	−5.35	−5.285	−5.94	2.48642	Hydrogen Bond	Conventional Hydrogen Bond
N:UNK1:H—A:GLU468:O	−5.595	−5.118	−11.749	2.18653	Hydrogen Bond	Conventional Hydrogen Bond
N:UNK1:H—A:LYS462:O	−5.462	−10.4225	−3.84	2.5903	Hydrogen Bond	Conventional Hydrogen Bond
N:UNK1:H—N:UNK1:O	−7.251	−6.2815	−3.388	2.26536	Hydrogen Bond	Conventional Hydrogen Bond
A:ALA1774—N:UNK1	−2.33283	−5.24483	−3.308	4.68248	Hydrophobic	Alkyl
N:UNK1:C—A:LYS462	−7.39817	−10.1332	−1.0585	3.93045	Hydrophobic	Alkyl

**Table 4:** Chemical interaction based on the interaction of Gentamicin within the binding pocket of SIPA1L1.

Name	XYZ: X	XYZ: Y	XYZ: Z	Distance	Category	Types
A:HIS484:HD1—N:UNK1:O	−6.0465	6.165	−2.399	2.92598	Hydrogen Bond	Conventional Hydrogen Bond
A:VAL895:HN—N:UNK1:O	−9.0935	6.457	0.3795	2.29179	Hydrogen Bond	Conventional Hydrogen Bond
N:UNK1:H—A:ASN457:O	−6.663	4.8475	2.488	2.04784	Hydrogen Bond	Conventional Hydrogen Bond
N:UNK1:H—A:VAL460:O	−6.666	2.162	1.832	2.28452	Hydrogen Bond	Conventional Hydrogen Bond
N:UNK1:H—A:THR892:O	−12.508	8.568	−4.787	2.14731	Hydrogen Bond	Conventional Hydrogen Bond
A:LYS797—N:UNK1	−8.48533	4.51433	−7.24017	3.77049	Hydrophobic	Alkyl
N:UNK1:C—A:PRO762	−6.743	10.0728	−5.02483	5.30008	Hydrophobic	Alkyl
N:UNK1:C—A:PRO762	−7.408	8.46733	−7.10683	4.03608	Hydrophobic	Alkyl
N:UNK1:C—A:LYS797	−7.92183	6.03483	−7.33317	3.92415	Hydrophobic	Alkyl

**Table 5:** Summary of GEO Datasets Used in the Meta-Analysis of Acne Vulgaris.

GEO Accession	Study Description	Sample Groups	Total Samples
GSE6475	Expression profiling of acne lesions and normal skin samples to identify baseline gene expression differences in Acne Vulgaris.	- 6 Acne lesion samples - 6 Normal skin from acne patients - 6 Normal skin from non-acne individuals	18
GSE10433	Gene expression analysis before and after 1-week isotretinoin treatment to identify early response genes.	- 6 Baseline (pre-treatment) samples - 6 post-treatment (1 week) samples	12
GSE11792	Gene expression profiling of skin samples before and after 8-week isotretinoin treatment to assess long-term transcriptional changes.	- 8 Baseline (pre-treatment) samples - 8 post-treatment (8 weeks) samples	16

## Data Availability

The datasets analyzed in this study are publicly available in the NCBI Gene Expression Omnibus (GEO) repository (https://www.ncbi.nlm.nih.gov/) under accession numbers **GSE6475**, **GSE10433**, and **GSE11792**.
